# Heterochromatin base pair composition and diversification in holocentric chromosomes of kissing bugs (Hemiptera, Reduviidae)

**DOI:** 10.1590/0074-02760160044

**Published:** 2016-10

**Authors:** Vanessa Bellini Bardella, Sebastián Pita, André Luis Laforga Vanzela, Cleber Galvão, Francisco Panzera

**Affiliations:** 1 Universidade Estadual Paulista, Instituto de Biociências, Departamento de Biologia, Rio Claro, SP, Brasil; 2 Universidad de la República, Facultad de Ciencias, Sección Genética Evolutiva, Montevideo, Uruguay; 3 Universidade Estadual de Londrina, Centro de Ciências Biológicas, Departamento de Biologia Geral, Londrina, PR, Brasil; 4 Instituto Oswaldo Cruz, Laboratório Nacional e Internacional de Referência em Taxonomia de Triatomíneos, Rio de Janeiro, RJ, Brasil

**Keywords:** Heteroptera, Triatominae, holocentric, chromosomes, CMA/DAPI banding, repetitive DNA

## Abstract

The subfamily Triatominae (Hemiptera, Reduviidae) includes 150 species of blood-sucking insects, vectors of Chagas disease or American trypanosomiasis. Karyotypic information reveals a striking stability in the number of autosomes. However, this group shows substantial variability in genome size, the amount and distribution of C-heterochromatin, and the chromosome positions of 45S rDNA clusters. Here, we analysed the karyotypes of 41 species from six different genera with C-fluorescence banding in order to evaluate the base-pair richness of heterochromatic regions. Our results show a high heterogeneity in the fluorescent staining of the heterochromatin in both autosomes and sex chromosomes, never reported before within an insect subfamily with holocentric chromosomes. This technique allows a clear discrimination of the heterochromatic regions classified as similar by C-banding, constituting a new chromosome marker with taxonomic and evolutionary significance. The diverse fluorescent patterns are likely due to the amplification of different repeated sequences, reflecting an unusual dynamic rearrangement in the genomes of this subfamily. Further, we discuss the evolution of these repeated sequences in both autosomes and sex chromosomes in species of Triatominae.

The subfamily Triatominae includes 150 species allocated to five tribes and 18 genera and belongs to the suborder Heteroptera (Galvão & de Paula 2014). These insects are mostly distributed in the New World and are vectors of Chagas disease, which affects approximately six to seven million people worldwide, mostly in Latin America (WHO 2016). In addition to their medical importance, triatomines are a biological model in chromosome segregation analysis because they harbour holocentric chromosomes (with diffuse or non-localised centromeres) (Pérez et al. 2000). Unlike other hemipteran groups, members of the subfamily Triatominae present high uniformity in their diploid chromosome number. Almost all of the 90 studied species have 20 autosomes (A), with only three exceptions (Panzera et al. 2010). Chromosome variation is almost exclusively caused by the occurrence of different systems of sex chromosomes (XY, X1X2Y and X1X2X3Y). Although there is an extensive karyotypic stability for autosomal complement, the genome size as well as the amount and distribution of some repetitive DNAs, such as C-heterochromatin, and the chromosome positions of 45S rDNA clusters are highly variable. These characteristics have not been reported previously in other organisms with holocentric chromosomes (Panzera et al. 2010, 2012). The main source of karyotype differentiation in Triatominae is the constitutive heterochromatin variation revealed by the C-banding technique. Constitutive heterochromatin is mainly composed of tandem (satellite DNA) and dispersed (transposable elements) repetitive DNA (Charlesworth et al. 1994). It is a key component in the structural and functional organisation of chromosomes. Heterochromatin is involved in various functions such as chromosome pairing, segregation, and even gene expression (Dimitri et al. 2009).

Heterochromatin variation in triatomines includes remarkable changes in the amount, size, chromosome location and behaviour of C-blocks during cell divisions (Panzera et al. 2010). The amount of heterochromatin varies over a wide range in the autosomal complement, from 45% (in *Triatoma delpontei*) to 0% in species of the matogrossensis and rubrovaria subcomplexes (Panzera et al. 2010). The size of heterochromatic blocks is also highly variable: they can be very tiny, as observed in *Rhodnius pallescens* (Gómez-Palacio et al. 2008), or they can constitute 80% of the entire chromosome, as in *T. nitida* (Panzera et al. 2010). Heterochromatic regions can be found in one, two or even in all autosomal pairs. Heterochromatic regions are generally located at one or both chromosomal ends, but in rare cases, interstitial bands are also observed (Panzera et al. 2014). Regarding sex chromosomes, all triatomine species show an almost entirely heterochromatic Y chromosome, while only a few species exhibit a C-heterochromatic X chromosome (Panzera et al. 2010, 2014).

Considering the high diversity in the amount and distribution of C-heterochromatin in the autosomes and sex chromosomes of triatomines, there are few studies exploring the base composition of heterochromatic regions. To date, the heterochromatin base-pair richness has been studied in only four *Triatoma* species (Pérez et al. 2000, Bardella et al. 2010, 2014a). The aim of the present study was to compare the karyotypes of 41 species from six different genera according to the occurrence and distribution of DAPI/CMA bands, thereby allowing a better characterisation of heterochromatic regions in terms of their relative enrichment with A+T or G+C base pairs, respectively. With this strategy, we can suggest a broad overview of the evolution of these repeated sequences both in autosomes and in sex chromosomes in the subfamily Triatominae.

## MATERIALS AND METHODS

*Biological material* - Table summarises the geographic origin, male diploid chromosome number and fluorescent banding results for the material analysed here. No specific permissions were required for the insect collections performed in this paper, and this study did not involve endangered or protected species. Chromosome preparations for C-CMA/DAPI banding were made from males and females of 41 triatomine species from the two principal tribes of the subfamily, Rhodniini and Triatomini, which include more than 90% of the 150 recognised species. For the Rhodniini tribe, which includes 13 species in two genera, we analysed two species: *Rhodnius prolixus* and *R. pallescens*. For Triatomini tribe we studied species from the following five genera: *Dipetalogaster* (one sp.), *Eratyrus* (one sp.), *Mepraia* (two spp.), *Panstrongylus* (five spp.) and *Triatoma* (30 spp.). By far, the most numerous genus is *Triatoma*, with 73 species. Within this genus, we studied the three main clades or groups: (a) the Rubrofasciata Group (from Central and North America and Old World species), (b) the Dispar Group (west of the Amazon Region), and (c) the Infestans Group (from south and east of the Amazon Region). Within each clade, we analysed various complexes and subcomplexes, according to the subdivisions proposed by Schofield and Galvão (2009) with modifications recently proposed by Pita et al. (2016).

**TABLE:** Geographic origin, diploid chromosome number and fluorescent results of Triatominae species currently analysed

Species	Male diploid number (2n)	Autosomal CMA+	Autosomal DAPI+	Fluorescence in X chromosome	Fluorescence in Y chromosome	Geographic origin^a^
Genus *Rhodnius*						
*R. prolixus* (Fig. 1A-B)	20A+XY	No	No	No	No	Guatemala, Quezaltenango, Las Palmas. D. Insectary CDC (USA)
*R. pallescens* (Fig. 1C-D)	20A+XY	1II with dot in 1 end	Some II with dots in 1 end	No	No	Colombia, Magdalena, Santa Marta, Mendihuaca, P.
Genus *Dipetalogaster*						
*D. maxima* (Fig. 1E-F)	20A+XY	1II with dot in 1 end	No	No	DAPI^+^/CMA^-^	Mexico, Baja California, La Paz. S.
Genus *Eratyrus*						
*E. cuspidatus* (Fig. 1G-H)	20A+X_1_X_2_Y	No	1 II with dot in 1 end	No	DAPI^+^ with CMA dot	Colombia, Sucre, San Onofre. S.
Genus *Mepraia*						
*M. spinolai* (Fig. 1I-J)	20A+X_1_X_2_Y	10 II with block in 2 ends	No	X_1_ and X_2_ with CMA dot in 2 ends	DAPI^+^/CMA^-^	Chile, Atacama Region, Inca Oro, P.
*M. gajardoi*	20A+X_1_X_2_Y	Some II with terminal dots	No	No	DAPI^+^/CMA^-^	Chile, Arica Region, Military History Museum, Arica. S.
Genus *Panstrongylus*						
*P. herreri*	20A+X_1_X_2_Y	10 II with block in 1 or 2 ends	Co-located with CMA regions in 10 II	No	DAPI^+^/CMA^-^	Peru, Amazonas, Utcubamba, P.
*P. chinai* (Fig. 1K-L)	20A+X_1_X_2_Y	10 II with block in 1 or 2 ends	Co-located with CMA regions in 10 II	No	DAPI^+^/CMA^-^	Ecuador, Loja, Limones, P.
*P. rufotuberculatus*	20A+X_1_X_2_Y	10 II with block in 1 or 2 ends	Co-located with CMA regions in 10 II	No	DAPI^+^/CMA^-^	Colombia, AN, Amalfi. P. / Colombia, Guajira, Gurnake, P.
*P. megistus* (Fig. 1M-N)	18A+X_1_X_2_Y	9 II with dots in 2 ends	No	No	DAPI^+^/CMA^-^	Brazil, MG, P. LNIRTT.
*P. geniculatus* (Fig. 1O-P)	20A+X_1_X_2_Y	No	No	X_1_: No. X_2_: DAPI dot	DAPI^+^/CMA^-^	Colombia, AN, Amalfi, P./ Venezuela, LNIRTT.
Genus *Triatoma*						
Group dispar	Complex	Dispar				
*T. boliviana* (Fig. 2A-B)	20A+XY	No	No	No	DAPI^+^/CMA^-^	Bolivia, La Paz, Muñecas, Vilaque. P.
*T. carrioni*	20A+XY	2-3 II with dots in 1 end	Co-located with CMA regions in 2-3 II	No	DAPI^+^/CMA^-^	Peru, Piura, Ayacuiba. S
Group Rubrofasciata	Complex	Protracta				
*T. rubrofasciata* (Fig. 2C-D)	22A+X_1_X_2_Y	11 II with blocks in 2 ends	Co-located with CMA regions in 11 II	No	DAPI^+^/CMA^-^	Vietnam, Hanoi, P.
*T. nitida* (Fig. 2E-F)	18A+X_1_X_2_Y	2 II almost entirely. Other II with dot	Co-located with CMA regions in 2 II	No	DAPI^+^/CMA^-^	Guatemala, Quiché, Zacualpa, D/ Guatemala, Chiquimula, El Sillón, P.
*T. barberi*	20A+X_1_X_2_Y	10 II with blocks in 2 ends	Co-located with CMA regions in 10 II	X_1_: DAPIdot plus CMA dot. X_2_: No.	DAPI^+^/CMA^-^	Mexico, Oaxaca, Etlo, P.
*T. protracta* (Fig. 2G-H)	20A+X_1_X_2_Y	10 II with blocks in 2 ends	Co-located with CMA regions in 10 II	X_1_: DAPIdot plus CMA dot. X_2_: No.	DAPI^+^/CMA^-^	USA, California, Monte Diablo. LNIRTT.
Complex Lecticularia						
*T. lecticularia* (Fig. 2I-J)	20A+XY	10 II with blocks in 2 ends	Co-located with CMA regions in 10 II	CMA dot	DAPI^+^/CMA^-^	USA, Oklahoma, Valkiria. LNIRTT.
Complex Phyllosoma						
*T. bassolsae* (Fig. 2K-L)	20A+X_1_X_2_Y	1 II with dot in 1 end	Co-located with CMA region in 1 II	No	DAPI^+^/CMA^-^	Mexico, Puebla. P.
*T. longipennis*	20A+X_1_X_2_Y	No	No	No	DAPI^+^/CMA^-^	Mexico, Zacatecas, Apozol, P.
*T. ryckmani* (Fig. 2M-N)	20A+X_1_X_2_Y	No	1 II with dot in 1 end	X_1_ and X_2_ with DAPI dots	DAPI^+^/CMA^-^	Guatemala, El Progreso, P.
*T. dimidiata* (Fig. 2O-P)	20A+X_1_X_2_Y	No	10 II with dots in 2 ends	No	DAPI^+^/CMA^-^	Mexico, Oaxaca, Nopala, P. Guatemala, Jutiapa, Carrizal, D.
Species	Male diploid number (2n)	Autosomal CMA+	Autosomal DAPI+	Fluorescence in X chromosome	Fluorescence in Y chromosome	Geographic origin^a^
Group Infestans						
Complex Infestans	Subcomplex	Infestans				
*T. delpontei* (Fig. 3A-B)	20A+XY	10 II with subterminal blocks in 1 end	Adjacent to CMA regions	DAPI dot, CMA dot	DAPI^+^/CMA^-^	Bolivia, ST, Tita, S. Argentina, SA, Rivadavia, S.
*T. platensis*	20A+XY	2-3 II with subterminal blocks in 1 or 2 ends	Adjacent to CMA regions	DAPI dot, CMA dot	DAPI^+^/CMA^-^	Uruguay, Paysandú, S.
T. infestans melanosoma* (non-Andean lineage)	20A+XY	3 II with subterminal blocks in 1 or 2 ends	Adjacent to CMA regions	DAPI dot	DAPI^+^/CMA^-^	Origin: ND. UNESP and LNIRTT.
T. infestans** (Andean lineage)	20A+XY	3-4 II with subterminal blocks in 1 or 2 ends	7-10 II with blocks in 1 or 2 ends. In 3-4 II adjacent to CMA regions	DAPI dot in both ends	DAPI^+^/CMA^-^	Peru: Andean regions. UNESP.
Subcomplex Maculata						
*T. maculata*	20A+XY	No	10 II with dots in 2 ends	No	DAPI^+^/CMA^-^	Brazil, RO. LNIRTT.
Subcomplex Sordida						
*T. garciabesi*	20A+XY	No	No	No	DAPI^+^/CMA^-^	Bolivia, ST, Izozog, S./ Argentina, SA, Rivadavia, LNIRTT.
T. matogrossensis*	20A+XY	No	No	No	DAPI^+^ plus CMA dot	Origin: ND. UNESP and LNIRTT.
*T. sordida* Argentina (Fig. 3C-D)	20A+XY	No	No	Full CMA^+^	DAPI^+^ plus CMA dot	Argentina, CO, San Luis del Palmar, P.
*T. sordida sensu stricto* (Fig. 3E-F)	20A+XY	10 II with small blocks in 1 or 2 ends	Co-located with CMA regions in 10 II	CMA dot	DAPI^+^/CMA^-^	Brazil, MG, M Claros and SJ do Povo, P. LNIRTT./Paraguay, Pte Hayes, Jope, P./ Bolivia, ST, Cotoca and Izozog, D.
*T. sordida La Paz* (Fig. 3G-H)	20A+XY	3-4 II with blocks in 1 or 2 ends	7 II with blocks in 1 or 2 ends. In 3-4 II co-located with CMA regions	No	DAPI^+^/CMA^-^	Bolivia, La Paz, Inquisivi, D.
*T. vandae*	20A+XY	No	No	No	DAPI^+^/CMA^-^	Brazil, MT, Rondópolis. LNIRTT.
Subcomplex Pseudomaculata						
*T. costalimai*	20A+XY	1 II with dot in 1 end	No	No	DAPI^+^/CMA^-^	Brazil, GO, Posse, S. LNIRTT.
*T. guazu* (Fig. 3I-J)	20A+XY	1 II with dot in 1 end	No	No	DAPI^+^/CMA^-^	Brazil, MT, Barra das Garças, LNIRTT.
*T. wygodzinsky* (Fig. 3K-L)	20A+XY	No	No	No	DAPI^+^/CMA^-^	Brazil, SP, ES do Pinhal, P. LNIRTT.
Subcomplex Rubrovaria						
T. rubrovaria*	20A+XY	No	No	No	DAPI^+^/CMA^-^	Origin: ND. UNESP and LNIRTT.
*T. rubrovaria*	20A+XY	No	No	No	DAPI^+^/CMA^-^	Uruguay, Artigas, S.
*T. carcavalloi*	20A+XY	1 II with dot in 1 end	No	No	DAPI^+^/CMA^-^	Brazil, RGS, S. LNIRTT.
*T. guasayana*	20A+XY	No	No	No	DAPI^+^/CMA^-^	Bolivia, ST, Izozog, S.
*T. klugi*		No	No	No	DAPI^+^/CMA^-^	Brazil, RGS, S. LNIRTT.
Subcomplex Brasiliensis						
T. brasiliensis*	20A+XY	No	No	No	DAPI^+^/CMA^-^	Origin: ND. UNESP and LNIRTT.
*T. brasiliensis brasiliensis* (Fig. 3M-N)	20A+XY	10 II with terminal blocks in 2 ends	3-4 II with subterminal dots adjacent to CMA regions	No	DAPI^+^/CMA^-^	Brazil, CE, Novo Oriente, D. Brazil, PE, Terra Nova, P.
*T. juazeriensis*	20A+XY	10 II with terminal blocks in 2 ends	3-4 II with subterminal dots adjacent to CMA regions	No	DAPI^+^/CMA^-^	Brazil, BA, Juazeiro. LNIRTT.
*T. sherlocki*	20A+XY	10 II with terminal blocks in 2 ends	3-4 II with subterminal dots adjacent to CMA regions	No	DAPI^+^/CMA^-^	Brazil, BA, P. LNIRTT.
Species without group assigned						
*T. vitticeps* (Fig. 3O-P)	20A+X_1_X_2_X_3_Y	No	No	X_1_: DAPI dot, 2Xs: No	DAPI^+^ plus CMA dot	Brazil, RJ and ES, LNIRTT.

Species are grouped according to the subdivisions proposed by Schofield and Galvão (2009) with modifications recently proposed by Pita et al. (2016). A: autosomes; II: bivalents; ND: not determined; P: peridomiciliary; D: domiciliary; S: sylvatic. UNESP: Universidade Estadual Paulista Júlio de Mesquita Filho, Faculdade de Ciências Farmacêuticas, Laboratório de Parasitologia, Araraquara, SP, Brasil; LNIRTT: Laboratório Nacional e Internacional de Referência em Taxonomia de Triatomíneos, Instituto Oswaldo Cruz, Rio de Janeiro, RJ, Brazil; NA: Antioquia; BA: Bahia; CE: Ceará; CO: Corrientes; ES: Espírito Santo; GO: Goiás; MT: Mato Grosso; MG: Minas Gerais; MS: Mato Grosso do Sul; PE: Pernambuco; RGS: Rio Grande do Sul; RJ: Rio de Janeiro; RO: Roraima; SA: Salta; SE: Santiago del Estero; SP: São Paulo; ST: Santa Cruz. *: data from Bardella et al. (2010); **: data from Bardella et al. (2014a).

*Chromosome preparation and banding procedures* - Gonads (testes and ovaries) were fixed in ethanol-acetic acid (3:1) and softened in a 45% aqueous solution of acetic acid.

We applied a C-banding technique on squashed preparations according to Panzera et al. (2010). Then, the slides were treated with fluorochromes: 0.5 mg/mL chromomycin A_3_ (CMA) for 1.5 h and 2 μg/mL 4’-6-diamidino-2-phenylindole (DAPI) for 30 min. Slides were mounted with a medium composed of glycerol/McIlvaine buffer, pH 7.0 (1:1, v:v), plus 2.5 mM MgCl_2_ and preserved at -20ºC. All slides were treated with both fluorochrome dyes (first CMA and then DAPI) in order to minimise the false-positive DAPI signals reported by Barros e Silva and Guerra (2010).

In all triatomine species, during meiotic prophase until diakinesis, the sex chromosomes always appear heteropycnotic with conventional staining and fluorescent banding. However, in metaphase plates, the sex chromosomes may have DAPI- or CMA-positive regions or similar fluorescent staining of the rest of the autosomes. The high fluorescent staining during the first meiotic prophase is consistent with the allocycly of these chromosomes during male meiosis, and it probably reflects a high degree of chromatin condensation rather than differences in base composition, although the latter has been suggested in other hemipteran species (Rebagliati et al. 2003). For this reason, we established the fluorescent patterns of sex chromosomes by analysing metaphases of both meiotic divisions and, when possible, in gonial mitosis. For autosomes, diplotene and early meiotic prophase were also analysed to detect small fluorescent regions that were not observed during metaphase due to high chromosome condensation. The pattern of fluorescence for each species was determined based on the chromosomal analyses of at least two individuals.

*Microscopy and imaging* - Slides were analysed under a Nikon Eclipse 80i epifluorescence microscope. Images were obtained with a Nikon DS-5 Mc-U2 digital cooled camera using Nikon Nis Elements 3.1 Advanced Research software and processed with Adobe Photoshop® software.

## RESULTS

We observed a striking variation in fluorescent banding of the 41 analysed species. The table and figures show the detailed results by species. Considering the complexity and diversity of the observed fluorescent profiles, we present the results for the autosomes and for each sex chromosome (Xs and Y) separately.

*Autosomal chromatin by fluorescent banding* - In triatomines, all autosomal C-heterochromatic regions always exhibited fluorescence staining, and we identified five fluorescence patterns.

**Fig. 1:**
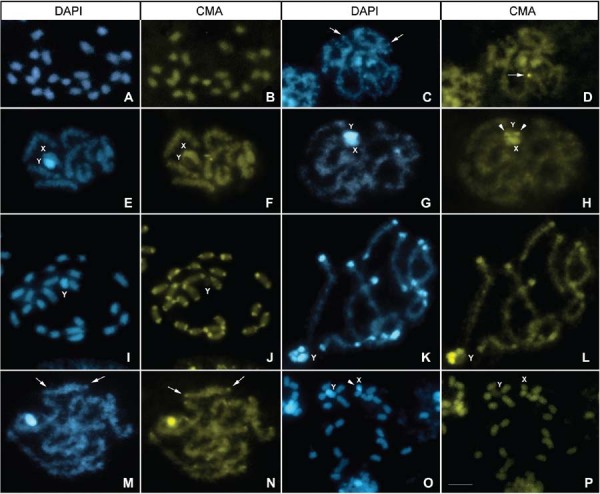
: fluorescent banding in several genera of Triatominae species, excepting *Triatoma*. (A-B) *Rhodnius prolixus*: spermatogonial prometaphase (2n = 22) without fluorescent signals in autosomes and sex chromosomes; (C-D) *R. pallescens*: diffuse stage showing DAPI and CMA autosomal dots (arrows); (E-F) *Dipetalogaster maxima*: pachytene with a CMA dot in one bivalent. The Y chromosome was completely CMA-/DAPI+, while the X chromosome did not exhibit fluorescent signals, similar to the case observed in other species; (G-H) *Eratyrus cuspidatus*: diffuse stage with a DAPI+ dot in one bivalent. The Y chromosome was entirely DAPI+ with a terminal CMA region (arrowhead); (I-J) *Mepraia spinolai*: spermatogonial prometaphase (2n = 23) showing 20 autosomes with CMA blocks in both chromosomal ends but no DAPI signal. The Y chromosome was CMA-/DAPI+, whereas both X chromosomes showed a terminal CMA band; (K-L) *Panstrongylus chinai*: early diplotene. Ten autosomal bivalents exhibited terminal co-localised CMA+/DAPI+ regions in both chromosomal ends; (M-N) *P. megistus*: diffuse stage with CMA+ dots in several bivalents (arrows); (O-P) *P. geniculatus*: spermatogonial prometaphase (2n = 23) without autosomal fluorescent regions. One X chromosome showed a DAPI+ dot (arrowhead). Bar: 10 μM.

**Fig. 2:**
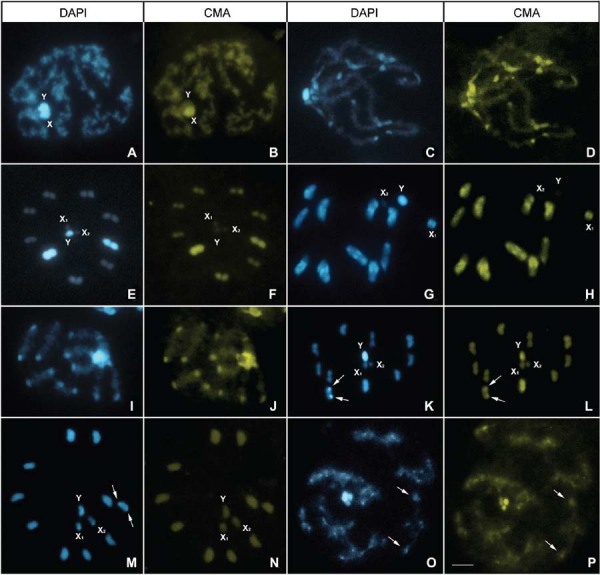
: fluorescent banding in several *Triatoma* species (Dispar and Rubrofasciata groups). (A-B) *T. boliviana*: diffuse stage without fluorescent signals in autosomes, and only the Y chromosome was completely DAPI stained; (C-D) *T. rubrofasciata*: diffuse stage showing all autosomes with CMA+/DAPI+ regions co-localized; (E-F) *T. nitida*: metaphase II with two half-bivalents showing almost entirely co-localised CMA/DAPI regions. The Y chromosome was entirely DAPI+, while both X chromosomes showed no fluorescent signals; (G-H) *T. protracta*: metaphase I with all bivalents showing terminal co-localised CMA+/DAPI+ regions. The Y chromosome was DAPI+, while the larger X chromosome (X1) showed only CMA+ and DAPI+ dots, and the X2 chromosome showed no fluorescence signals. (I-J) *T. lecticularia*: pachytene with all autosomes with co-localised CMA+/DAPI+ regions; (K-L) *T. bassolsae*: metaphase II showing one half-bivalent with a co-localised CMA+/DAPI+ dot (arrows); (M-N) *T. ryckmani*: metaphase II showing one half-bivalent with a DAPI+ dot (arrows) and both X chromosomes have terminal DAPI+ dots; (O-P) *T. dimidiata*: diplotene stage showing all bivalents with co-localised CMA+/DAPI+ dots (arrows). Bar: 10 μM.

**Fig. 3:**
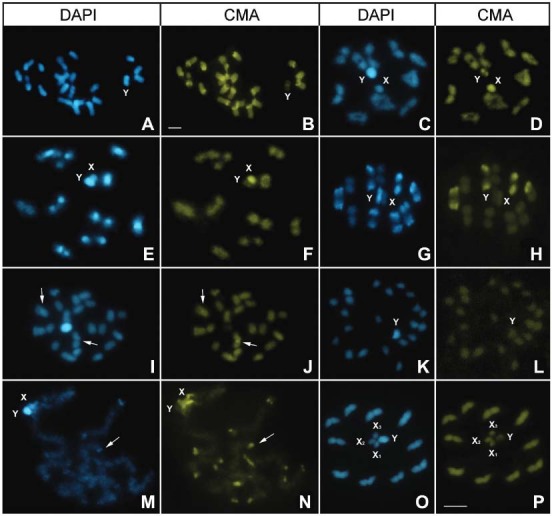
: fluorescent banding in several *Triatoma* species from South America (Infestans group). (A-B) *T. delpontei*: spermatogonial prometaphase (2n = 22) with all autosomes showing a large fluorescent block composed of two sub-regions: a terminal DAPI+ region and a sub-terminal CMA+ region, in only one chromosomal end. (C-D) *T. sordida Argentina*: late diplotene showed no fluorescent bands on their ten autosomal pairs. The Y chromosome is DAPI+ with CMA+ dots, while the X chromosome is DAPI-negative but positive for CMA staining; (E-F) *T. sordida sensu stricto*: last diplotene. All bivalents showed CMA+/DAPI+ regions co-localised. The Y chromosome was DAPI+ and negative for CMA, while the X chromosome had the inverse staining pattern; (G-H) *T. sordida La Paz*: metaphase I with seven bivalents showed DAPI+ blocks, four of them also with CMA+ staining. The Y chromosome is entirely DAPI+, while the X chromosome had no fluorescent regions; (I-J) *T. guazu*: Spermatogonial prometaphase (2n = 22) without autosomal fluorescent bands, except one autosomal pair with an interstitial CMA+ band (arrows); (K-L) *T. wygodzinsky*: spermatogonial prometaphase without autosomal fluorescent bands; (M-N) *T. brasiliensis*: pachytene stage. All autosomes showed terminal CMA+ dots on both chromosomal ends. Some bivalents also presented sub-terminal DAPI+ dots, adjacent to CMA+ regions (arrows); (O-P) *T. vitticeps*: metaphase II showing all autosomes without fluorescent bands. The Y chromosome was totally DAPI+ with one terminal CMA+ dot. Furthermore, one X chromosome exhibited a DAPI+ dot, whereas the other two X chromosomes showed no fluorescent staining. Bar: 10 μM.

*Pattern 1* - *Autosomal euchromatic regions without CMA/DAPI bands (* Figs 1A-B, 2A-B, 3C-D, K-L, O-P). This is the most frequent pattern in triatomine species without autosomal C-heterochromatin, *e.g.*, *R. prolixus*, *T. boliviana*, *T. garciabesi*, *T. guasayana*, *T. klugi*, *T. longipennis*, *T. matogrossensis*, *T. rubrovaria*, *T. sordida Argentina*, *T. vandae*, *T. vitticeps* and *T. wygodzinsky*) (Table).

*Pattern 2* - *Autosomal C-heterochromatic regions with co-localisation of DAPI* ^*+*^ *and CMA* ^*+*^ *fluorescence* (Figs 1K-L, 2O-P, 3C-H). This is the most frequent pattern observed among triatomine species with autosomal C-heterochromatin, being detected in several *Panstrongylus* species as well as in numerous species from the three main groups of *Triatoma*. The number and size of the fluorescent regions are variable in each triatomine species, and they are detailed in the table.

*Pattern 3* - *Autosomal C-heterochromatin regions exclusively DAPI* ^*+*^. This pattern was observed in autosomes of *R. pallescens*, *E. cuspidatus*, *T. bassolsae*, *T. ryckmani*, *T. maculata* (Figs 1C-D, G-H, 2M-P) and some *T. infestans* (Andean lineage) and *T. sordida La Paz* (Fig. 3G-H).

*Pattern 4* - *Autosomal regions exclusively CMA* ^*+*^ *.* Detected in *Mepraia* species (Fig. 1I-J), *P. megistus* (Fig. 1M-N) and brasiliensis subcomplex species (Fig. 3M-N). Furthermore, several species exhibited one autosomal pair with CMA dots (Figs 1E-F, 2K-L, 3I-J) (*D. maxima*, *T. bassolsae*, *T. costalimai*, *T. guazu* and *T. carcavalloi*). In these species, except for *D. maxima*, the autosomal CMA regions are associated with the locations of ribosomal genes.

*Pattern 5* - *Autosomal C-heterochromatin region constituted by two adjacent sub-regions: a terminal DAPI* ^*+*^ *and a subterminal CMA* ^*+*^. This pattern was only observed in the three infestans subcomplex species (Fig. 3A-B). In brasiliensis subcomplex species, some autosomes presented an inverse pattern, *i.e.*, terminal CMA regions with subterminal DAPI bands (Fig. 3M-N).

Despite the identification of these five autosomal fluorescence patterns, some species presented autosomes with more than one of the patterns described above. For example, *T. sordida La Paz* (Fig. 3G-H), species of the brasiliensis subcomplex (Fig. 3M-N) and *T. infestans* present at least two types of fluorescent profiles in their autosomes.

*X chromosome by fluorescent banding* - Five fluorescent patterns were observed in the X chromosomes (Table). Most species showed euchromatic X chromosomes without fluorescent bands (30 species), with unstained chromatin similar to that observed in the autosomal euchromatin. Undifferentiated X chromosomes were observed in species with XY or X_1_X_2_Y sex systems among all genera studied (Figs 1-3). The other four patterns are associated with C-heterochromatin and involved the presence of different fluorescence bands, and they are as follows: (i) CMA^+^ bands in both chromosomal ends of X_1_ and X_2_ chromosomes in *M. spinolai* (Fig. 1I-J); (ii) DAPI^+^ bands in one chromosomal end of X_1_ and X_2_ chromosomes in *T. ryckmani* (Fig. 2M-N); (iii) CMA^+^ with a DAPI^+^ dot in a larger X chromosome of *T. protracta* and *T. barberi* (Fig. 2G-H); (iv) one X chromosome with a DAPI^+^ band in one end and a CMA^+^ dot in the other chromosomal end (*P. geniculatus* and *T. platensis*) (Fig. 1O-P).

*Y chromosome by fluorescent banding* - Two types of Y chromosomes were observed (Table). All Triatomini tribe species showed a Y chromosome that was entirely DAPI^+^ (Figs 1E-P, 2, 3), while in Rhodniini species, the Y chromosome did not exhibit fluorescent signals (Fig. 1A-D). In four species of the Triatomini tribe (*E. cuspidatus*, *T. sordida* Argentina, *T. matogrossensis* and *T. vitticeps*), the DAPI^+^ Y chromosome also included a CMA^+^ region.

## DISCUSSION

The results presented here clearly show that the high chromosome variability displayed by C-banding in triatomines (for review see Panzera et al. 2010) is significantly increased with the application of fluorescent dyes. This technique allows a clear discrimination of the heterochromatic regions classified as similar by C-banding, thus constituting a new chromosome marker with taxonomic and evolutionary significance. An illustrative example is *T. protracta* and *M. spinolai*, two species exhibiting similar number, size and chromosome location of C-heterochromatic regions. However, the first species exhibited co-localised AT-GC rich regions, while the second species showed GC-rich bands exclusively (Figs 2G-H, 1I-J, respectively).

Reports on heterochromatin characterisation by DNA binding fluorochromes in Heteroptera are scarce and fragmentary, involving a small number of species from different families (Grozeva et al. 2004, Bressa et al. 2005, Chirino et al. 2013, Bardella et al. 2014b, c). Thus, its application as a taxonomic marker has not been successfully implemented. This technique has only recently been applied to differentiate cryptic species in the genus *Macrolophus* (Miridae) (Jauset et al. 2015).

This paper represents the most extensive C-fluorescence banding analyses applied to an insect group with holocentric chromosomes. We identified five autosomal fluorescent patterns in triatomines, some of them previously reported in other heteropteran species (Bressa et al. 2005, Bardella et al. 2014b, c, Bansal & Kaur 2015). However, patterns four and five are uncommon in Heteroptera. Pattern four was recently described in the coreid *Holhymenia histrio*, but most of its regions are in intercalary position (Bardella et al. 2014b). Pattern five (and its inverse) has not been described in any other insect group (Fig. 3A-B, M-N).

Each fluorescent pattern reflects the presence of distinctive and specific sequences that compose the repetitive DNA. Furthermore, within the same pattern, different DNA repeats are likely to be involved, as observed in *T. infestans* (Bardella et al. 2014a). The diverse fluorescent patterns are probably due to the amplification of different repeated sequences, which reflects an extraordinary dynamic in the genomes of the Triatominae, never observed before in insects with holocentric chromosomes. This phenomenon contrasts with that observed in other heteropteran groups, in which each subfamily presents few heterochromatin types (Bressa et al. 2005, Bardella et al. 2014b).

Despite the striking variability in the fluorescent patterns, we observed that closely related species present similar compositions of A-T and/or C-G regions. For example, all species of the infestans subcomplex exhibited the same autosomal fluorescent profile (autosomal pattern five) (Fig. 3A-B, Table). Similar phenomena are observed among *Mepraia* species, several *Panstrongylus* species, and in the brasiliensis, rubrovaria and protracta subcomplexes. At the evolutionary level, the occurrence of similar DNA repeats among closely related species suggests a common ancestor having a particular satellite DNA library. This feature is recurrent in related species and has been reported in different insect groups such as Coleoptera (Pons et al. 2004) and Orthoptera (Yoshimura et al. 2006). In triatomines, a similar process was suggested in infestans subcomplex species by GISH studies (Pita et al. 2014).

*Diversity in X chromosome heterochromatin composition* - Similar to other hemipteran groups, most triatomine species (30 out of 41 species) showed euchromatic X chromosomes without fluorescent bands. However, four fluorescent patterns on the X chromosomes were detected, involving species with XY, X_1_X_2_Y and X_1_X_2_X_3_Y sex systems from three different genera (Figs 1-3). This paper reveals an extensive variability on the X chromosomes not reported in other heteropteran groups. Only in the family Coreidae have some variations on the X chromosomes been described (Bardella et al. 2014b). Considering that the heterochromatic X chromosome is observed in evolutionarily distant triatomine species involving different sex mechanisms, its occurrence is probably due to convergent evolution. Therefore, the ancestral X chromosome should have been similar to the euchromatic autosomes without C-heterochromatic regions or fluorescent banding.

*Variation in Y chromosome heterochromatin composition* - All Triatominae species currently analysed by conventional C-banding, approximately 90 species, showed a heterochromatic Y chromosome. However, fluorescent banding on Y chromosomes reveals a clear differentiation between the tribes Triatomini and Rhodniini. All species of Triatomini (39 species of five different genera, Table) showed an entirely DAPI^+^ Y chromosome, revealing that this chromosome is composed of AT-rich repeated sequences (Figs 1E-P, 2, 3). This high similarity shared among the Y chromosomes of Triatomini species supports the idea that this chromosome, including the repeat sequences contained therein, is a tribe ancestral character (Pita et al. 2014). Only in four species that are not closely related (*E. cuspidatus*, *T. sordida* Argentina, *T. matogrossensis* and *T. vitticeps*) did the DAPI^+^ Y chromosome also include a CMA^+^ region, which coincides with the existence of ribosomal genes (Bardella et al. 2010, Panzera et al. 2012, 2015). Considering the low frequency of species with Y chromosomes carrying CMA^+^ regions and the presence of CMA^+^ regions in evolutionarily distant species, we can conclude that this trait is a derivative character, at least for the Triatomini tribe. In addition, as CMA^+^ Y chromosomes are correlated with the localisation of ribosomal genes, the same hypothesis may apply. In contrast, the Y chromosome in the Rhodniini tribe does not exhibit DAPI staining, probably indicating that this chromosome is composed of other repeated sequences (Fig. 1A-D). Although *R. pallescens* has ribosomal genes on both sex chromosomes (Pita et al. 2013), we do not observe CMA^+^ bands on them.

In summary, the similar fluorochrome composition of the Y chromosome in Triatomini tribe and their striking differentiation with Rhodniini species detected here are consistent with GISH results (Pita et al. 2014). This chromosomal change is likely a consequence of evolutionary differentiation between these two tribes and may be useful as a phylogenetic marker.

Contradictory hypotheses support Triatominae as a monophyletic (Hypša et al. 2002, Weirauch & Munro 2009, Patterson & Gaunt 2010), polyphyletic (Schofield & Galvão 2009) or paraphyletic group (Hwang & Weirauch 2012). According to the latter models, the Triatomini and Rhodniini tribes are derived from quite different reduviid subfamilies. Considering that the split between the Rhodniini and Triatomini tribes is the most ancient known divergence point within triatomine bugs (between 40 and 95 million years ago) (Justi et al. 2016, Patterson & Gaunt 2010, respectively), comparative molecular analyses of the Y chromosome among triatomines with putative/suspected sister reduviid species may help to clarify the monophyletic or polyphyletic origin of Triatominae subfamily. For example, *Zelurus femoralis*, belonging to a Reduviinae genus considered to be a monophyletic group with Triatominae and Stenopodainae (Weirauch & Munro 2009), presented a DAPI^+^ Y chromosome similar to that of the Triatomini tribe (Poggio et al. 2013).
